# Metabolic reprogramming of macrophages in chronic obstructive pulmonary disease

**DOI:** 10.3389/fimmu.2025.1684075

**Published:** 2025-11-26

**Authors:** Ziwen Qin, Wenjuan Liu, Chuanjun Huang, Wei Zhang

**Affiliations:** 1 The First Clinical Medical College, Shandong University of Traditional Chinese Medicine, Jinan, Shandong, China; 2 Department of Pulmonary and Critical Care Medicine, Yantaishan Hospital, Yantai, Shandong, China; 3 Department of Respiratory and Critical Care Medicine, Shandong Provincial Hospital Affiliated to Shandong First Medical University, Jinan, Shandong, China; 4 Department of Respiratory and Critical Care Medicine, Shandong University of Traditional Chinese Medicine Affiliated Hospital, Jinan, Shandong, China

**Keywords:** chronic obstructive pulmonary disease, alveolar macrophages, macrophage polarization, immunometabolic reprogramming, immunometabolism

## Abstract

Chronic obstructive pulmonary disease (COPD) is currently one of the major causes of death and hospitalization globally. Pulmonary inflammation and oxidative stress are considered important mechanisms underlying the disease. Recent studies have indicated that the metabolic processes of immune cells in COPD, notably alveolar macrophages (AMs), may undergo significant alterations, exhibiting distinct metabolic characteristics related to their functional state and polarization phenotype. This phenomenon is known as the immunometabolic reprogramming of macrophages. In this article, we review the polarization phenotype and metabolic characteristics of macrophages in COPD, as well as the mechanisms affecting macrophage metabolism, and discuss the potential significance of pathways targeting immunometabolism of AMs in the treatment of COPD.

## 1 Introduction

Chronic obstructive pulmonary disease (COPD) is a chronic inflammatory pulmonary disease marked by persistent airflow obstruction. Its heterogeneity is manifested in parenchymal destruction and airway remodeling, often accompanied by chronic bronchitis and emphysema, which collectively lead to a progressive and irreversible decline in lung function. COPD symptoms often encompass coughing, expectoration, dyspnea, and wheezing. Prolonged tobacco exposure, occupational exposure to particulate matter environments, or indoor air pollution may exacerbate chronic airway inflammation, intensifying clinical symptoms and hastening disease progression. Poorly controlled COPD may further progress to cor pulmonale or even respiratory failure (1). According to World Health Organization (WHO) data, COPD currently ranks as the fourth leading cause of death globally, causing 3.5 million deaths in 2021, which constitutes approximately 5% of the total global mortality (2). COPD is regarded as the consequence of dynamic gene-environment interactions; however, its specific pathophysiology remains inadequately understood. Widely recognized mechanisms include airway oxidative stress, inflammatory responses, and protease/antiprotease imbalance. Furthermore, the crucial role of macrophages in the pathogenesis of COPD has been frequently reported.

The respiratory tract is in direct contact with the external environment (3), and its immune system, particularly alveolar macrophages (AMs), plays an important role in maintaining immunological homeostasis. AMs exert crucial immune defense and surveillance functions by phagocytosing inhaled contaminants and pathogens (4, 5). In COPD, this delicate balance was disrupted, as evidenced by elevated macrophage levels in the bronchoalveolar lavage fluid (BALF) and damaged lung tissue of patients (6), which may coordinate the immune response (7) but also contributed to persistent airway inflammation and alveolar destruction. More critically, AMs in COPD patients exhibited significant phagocytic defects, which correlated with worse pulmonary function following bacterial challenges (8). These observations raise a pivotal question: why do AMs become dysfunctional despite their increased abundance? This shifts the research focus from cell numbers to the potential mechanisms regulating their functional state.

The core of this functional dysregulation might be due to macrophage immunometabolic reprogramming—an emerging and crucial research area. AMs exhibit heterogeneous phenotypes under pathological conditions (9). Various studies pointed out that the metabolic alterations in AMs enhanced the adaptability and uniqueness of their functional subsets (10, 11). Macrophages are traditionally categorized into classically activated (M1) and alternatively activated (M2) phenotypes (12), and their functional states may shaped by core metabolic pathways: M1 polarization is linked to aerobic glycolysis (the “Warburg effect”), driving pro-inflammatory mediator production, whereas M2 polarization relies on oxidative phosphorylation (OXPHOS) and the tricarboxylic acid (TCA) cycle to support anti-inflammatory and repair functions (13, 14). Consequently, alterations in macrophage metabolic patterns may dictate their inflammatory responses (13, 15). In the specific condition of COPD, the imbalance of this “metabolism-polarization-function” axis is particularly critical: M1-associated glycolysis promotes tissue destruction, while dysregulated M2-associated metabolic processes can lead to abnormal tissue repair and fibrosis. Prominent metabolic alterations in AMs may strongly correlate with disease severity in COPD patients (16), introducing the viewpoint that metabolic reprogramming may not as an epiphenomenon but as a crucial promoter of COPD pathophysiology, explaining why targeting this axis represents a promising new therapeutic strategy.

This review focuses on the phenotypes and function states of AMs associated with the pathogenesis of COPD, as well as the specific mechanisms underlying their heterogeneous immunometabolism patterns, referred to as metabolic reprogramming, hoping to be exploited to develop potential therapeutic ideas for COPD.

## 2 Macrophage function and metabolic characteristics

Macrophages are extensively present in various tissues throughout the body and therefore can be classified according to their anatomical location. Based on ontological origin, the macrophage populations existing in the lungs consist primarily of tissue-resident (TR-) AMs, monocyte-derived (MO-) AMs, and interstitial macrophages. Notably, TR-AMs, which originate from embryonic development, are continuously renewed within lung tissue. Circulating monocytes in the peripheral blood can be rapidly recruited to the sites of infection or injury during pulmonary inflammation and subsequently differentiate into MO-AMs (17–20). Studies of airway AMs in lung transplant patients have reinforced the key position of peripheral blood CD14+CD16- (classical) monocytes, which constitute 85% of the circulating monocyte pool, in the origin of pulmonary AMs (21–24). Furthermore, based on their activation state, human monocyte-derived macrophages (MDMs) or murine bone marrow-derived macrophages (BMDMs) cultured *in vitro* are categorized as M1 or M2 macrophages. These subsets exhibit distinct functional tendencies and metabolic characteristics, contributing to the inflammatory process of COPD through complex immune and metabolic mechanisms (25, 26).

### 2.1 Macrophage polarization and function

Macrophages eliminate pathogens through phagocytosis, initiate the innate immune response in the lung, while also orchestrating pro-inflammatory, anti-inflammatory, and tissue repair processes. Changes in local microenvironmental signals induce macrophage polarization into distinct phenotypes (27). Upon stimulation with lipopolysaccharide (LPS) and/or interferon-γ (IFN-γ), M1 macrophages highly expressed inducible nitric oxide synthase (iNOS) and pro-inflammatory cytokines to drive chronic inflammation. Besides, they also inhibited tumor growth via anti-angiogenic effects (28). However, interleukin (IL)-4-activated M2 macrophages mainly participate in inflammation suppression and tissue repair, facilitating pathological angiogenesis, organ fibrosis, tumor growth, and the progression of allergic and parasitic diseases (28).

In COPD patients who smoke, studies indicated a positive correlation between pulmonary function decline/disease severity and the dual polarization of M1 and M2 in AMs. This contrasted with healthy individuals, whose airway AMs predominantly remained in a non-polarized state (7). Similarly, an increase in both the total number of pulmonary macrophages and the M2/M1 phenotype ratio was observed in the COPD mouse model (29), highlighting the critical role of excessively polarized macrophages in COPD pathologies.

#### 2.1.1 M1 macrophages

M1 macrophage polarization, triggered by pathogens or pro-inflammatory cytokines, is central to the immune response in COPD. It can be induced in macrophages upon stimulation with bacterial endotoxin LPS, Th1 pro-inflammatory cytokines such as IFN-γ, tumor necrosis factor α (TNF-α), or granulocyte-macrophage colony-stimulating factor (GM-CSF). This polarization is characterized by the expression of specific markers, including MHC-II molecules, CD80, and CD86 on the cell surface, alongside a cytokine profile featuring high levels of IL-12 and IL-23, but low IL-10 (30). Functionally, polarized M1 macrophages generate abundant inflammatory mediators, including reactive oxygen species (ROS), iNOS, TNF-α, IL-6, IL-1β, and the chemokines chemokine (C-X-C motif) ligand 9 (CXCL9/MIG), CXCL10/IP-10, and chemokine (C-C motif) ligand 2 (CCL2/MCP-1). Through antigen presentation, they initiated Th1-type immunity, recruiting Th1 lymphocytes in response to pathogenic microorganisms (30, 31). In COPD, this M1-driven response may become dysregulated and contribute to disease pathogenesis (1).

While M1-secreted mediators initially help clear pathogens, persistent M1 activation may lead to cytotoxic tissue injury (32). Highly expressed iNOS produced elevated nitric oxide (NO) by inducing the activation of nuclear factor kappa-B (NF-κB). Together with ROS and sustained Th1 cell recruitment, they induced substantial damage to lung tissue due to their cytotoxic effects (33). Clinically, Bazzan et al. reported an increase in both M1 and M2 macrophage proportions in the lungs of COPD patients, correlating with smoking history and disease severity. Moreover, M1 polarization was more pronounced and specifically correlated with the severity of airflow obstruction, as measured by FEV1/FVC% (7), indicating the involvement of M1 hyperpolarization in Th1 immune inflammation of COPD. Cigarette smoke (CS) further shifted macrophages toward an M1-dominated state by upregulating Wnt family member 5a (Wnt5a) and suppressing anti-inflammatory peroxisome proliferator-activated receptor γ (PPARγ), thereby reinforcing pulmonary inflammation and COPD in human and mouse models (34). Thus, the metabolic and functional profile of M1 macrophages is not merely descriptive; it underlies the sustained inflammation, tissue injury, and disease progression observed in COPD.

#### 2.1.2 M2 macrophages

Upon induction by Th2 cytokines such as IL-4, IL-13, IL-10, and transforming growth factor-β (TGF-β), polarized M2 macrophages release anti-inflammatory factors like IL-1, IL-10, and TGF-β. This functional response, which also enhances the phagocytosis of apoptotic cells and collagen deposition, serves to protect the host from inflammatory damage and promote tissue healing (35).

During the initial phase of mycobacterium tuberculosis infection, the expression of IFN-γ and iNOS in the BALF of mice gradually increased, aligning with the observed trend of AM polarization, indicating M1-biased AM polarization. However, as the inflammation persisted, the level of M1 markers gradually reduced while the M2 markers IL-4 and arginase 1 (Arg1) secretion were substitutionally enhanced. These results indicated a dynamic polarization plasticity during inflammatory progression, as evidenced by the transition from M1-dominance in acute infection to M2-preference in chronic phases (36). In COPD, this pattern manifested as increased M2 polarization during middle and later stages, initially serving to maintain pulmonary homeostasis and mitigate the excessive inflammatory responses (37). However, chronic M2 activation becomes unadaptive. Chronic inflammatory stimulation in COPD leads to repeated processes of damage and repair in lung tissues, while emphysema, the main pathological manifestation of COPD, is closely linked to the tissue repair function of M2 AMs (25). *In vivo*, chronic pulmonary inflammation resulting from prolonged exposure to CS can induce the highly expressed CD206 and TGF-β in macrophages, driving the adaptive immune response of the M2 phenotype to participate in tissue repair (38). Various inflammatory mediators secreted by M2 in the lung tissues of COPD patients were reported, such as matrix metalloproteinase (MMP)-2, MMP-9, MMP-12, and cathepsin S, to cause lung parenchymal injury and eventually form emphysema, with the increase in M2 amount positively correlated with the emphysema severities (39–41). An animal study reported that PM2.5-induced M2 AM polarization upregulated the level of MMP12 via the IL-4/STAT6 pathway in mice (42), while another research indicated that IL-4-induced M2 interstitial macrophages (IMs), rather than AMs, appeared to be the major producer of MMP-12 in lungs of COPD mice (43) However, similarly, both of these studies found that M2 macrophages caused the dysfunction of the alveolar epithelial barrier and ultimately led to COPD progression, suggesting that MMP-12 secreted by M2 phenotype may play an important role in the formation of emphysema signs in COPD. *In vivo* and *in vitro*, M2-directed polarization related to the TGF-β/Smad pathway in COPD was observed (29, 44), with the M2/M1 proportions generally negatively correlated with the lung function of mice (29), demonstrating the potential significance of the polarization state biased towards M2 macrophages in COPD.

**Figure 1:**
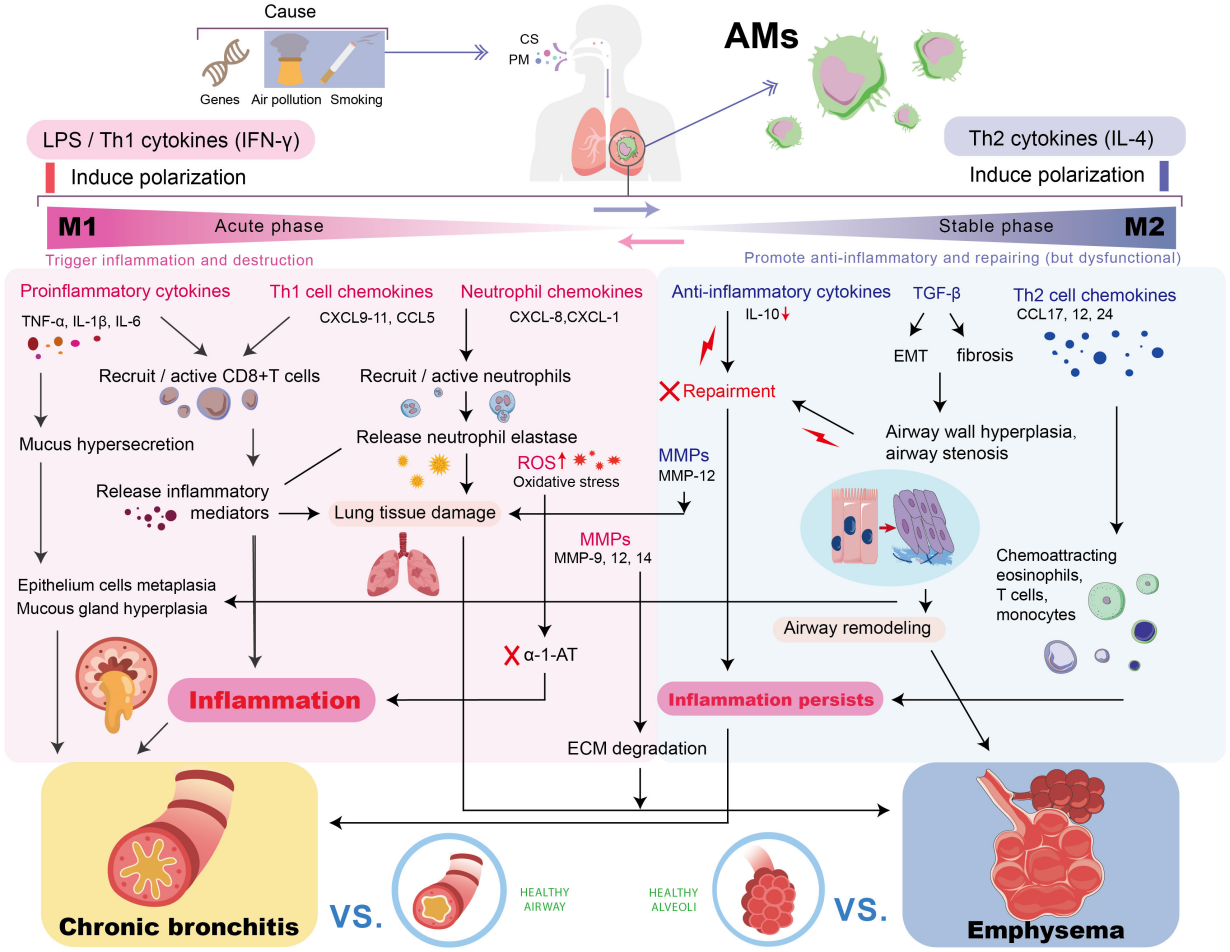
The Role of AMs in the pathogenesis of COPD. Chronic stimulation drives AMs to polarize into M1/M2 phenotypes. The sustained inflammation/damage mediated by M1 and the dysregulated repair/fibrosis of M2 jointly promote the key pathological processes in COPD, leading to persistent chronic inflammation, chronic bronchitis, and emphysema, ultimately resulting in progressive lung function decline. AM, alveolar macrophage; TNF-α, tumor necrosis factor-alpha; IL, interleukin; MMP, matrix metalloproteinase; ROS, reactive oxygen species; TGF-β, transforming growth factor-beta; IFN-γ, interferon-γ; CXCL, chemokine (C-X-C motif) ligand; CCL, C-C motif chemokine ligand; EMT, epithelial-mesenchymal transition; α-1-AT, α-1-antitrypsin; ECM, extracellular matrix.

As illustrated in **Figure 1**, AMs play an important role in the pathogenesis of COPD by driving chronic inflammation and tissue destruction. Additionally, **Table 1** summarizes the distribution of different macrophage phenotypes in COPD patients compared with non-COPD people, highlighting the characteristics in AM polarization of COPD.

**Table 1:** Macrophage phenotype distribution in COPD patients^#^.

Source	Method	Number	Object	Total Mø	M1	M2	Double-polarized (DP)	Non-polarized (NP)	Ref.
BALF	FC	18 (vs.10)	Marker COPD (vs.non-COPD)	Uncategorized	CD40+CD163− NS	CD40−CD163+ NS	CD40+CD163+ ↓	CD40−CD163− ↑	(45)
BALF	FC	8 (vs.17)	Marker COPD (vs.non-COPD)	Uncategorized	CD40+CD163− NS	CD40−CD163+ NS	CD40+CD163+ ↓	CD40−CD163− ↑	(46)
BALF	FC	47 (vs.30)	Marker COPD (vs.non-COPD)	Uncategorized	CD86+ ↑	CD206+ ↑	No detection	No detection	(47)
Lung tissues	IHC	38 (vs.25)	Marker COPD (vs.non-COPD)	CD68+ ↑(III/IV stage COPD)	No detection	CD163+, CD204+, CD206+ ↑(III/IV stage COPD)	No detection	No detection	(48)
Lung tissues	IHC	10 (vs.15)	Marker COPD (vs.non-COPD)	CD68+ ↑	iNOS+CD68+ ↓	CD206+CD68+ ↑	No detection	No detection	(49)
Lung tissues	IHC	23 (vs.30)	Marker COPD (vs.non-COPD)	Uncategorized	iNOS+ ↑	CD206+ ↑	iNOS+CD206+ ↑	iNOS-CD206- ↓	(7)
Lung tissues	IHC	7 (vs.16)	Marker COPD (vs.non-COPD)	CD68+ ↑	iNOS+CD68+ ↓	CD206+CD68+ ↑	No detection	No detection	(50)

^#^ ↑, increased; ↓, decreased; Mø, macrophages; FC, flow cytometry; IHC, immunohistochemistry.

However, it must be pointed out that this M1/M2 classification is a simplification of the complex functional state within the body. In chronic diseases such as COPD, macrophages may exhibit mixed phenotypes or a vague state between the two (46, 51). There are apparent contradictions in evidences regarding macrophage polarization in COPD, such as the debated impact of smoking on polarization subtypes (52). These discordances may arise from disease heterogeneity, variations in clinical stages, and differences in experimental systems. Supporting this complexity, studies by Bazzan et al. and He et al. reported associations of both mixed M1/M2 polarization and phenotype skewing with COPD severity (7, 29), challenging the traditional binary classification. The dynamic alterations between M1 and M2 phenotypes underline the necessity of a balanced response across disease stages for effective immunity and repair. Consequently, a full understanding of macrophage roles in COPD requires a shift in focus from rigid categorization towards dynamic equilibrium and condition-dependent functional states.

### 2.2 Metabolic events that occur in macrophages

The above-mentioned functional characteristics of macrophages are closely related to intracellular metabolic reprogramming. Under the stimulation of a specific microenvironment, the metabolic pattern switches between glycolysis and OXPHOS, involving the polarization tendency of M1/M2 macrophages (14). M1 macrophage metabolism mainly shifts to glycolysis, the pentose phosphate pathway (PPP), and fatty acid (FA) synthesis to fulfill their ATP demands (10, 53, 54). Meanwhile, the functions of the TCA cycle (Krebs cycle) and OXPHOS are compromised, accompanied by the downregulated fatty acid oxidation (FAO) (10, 55, 56). Nevertheless, the M2 type mainly relies on the full TCA cycle, OXPHOS, and FAO (54).

**Figure 2:**
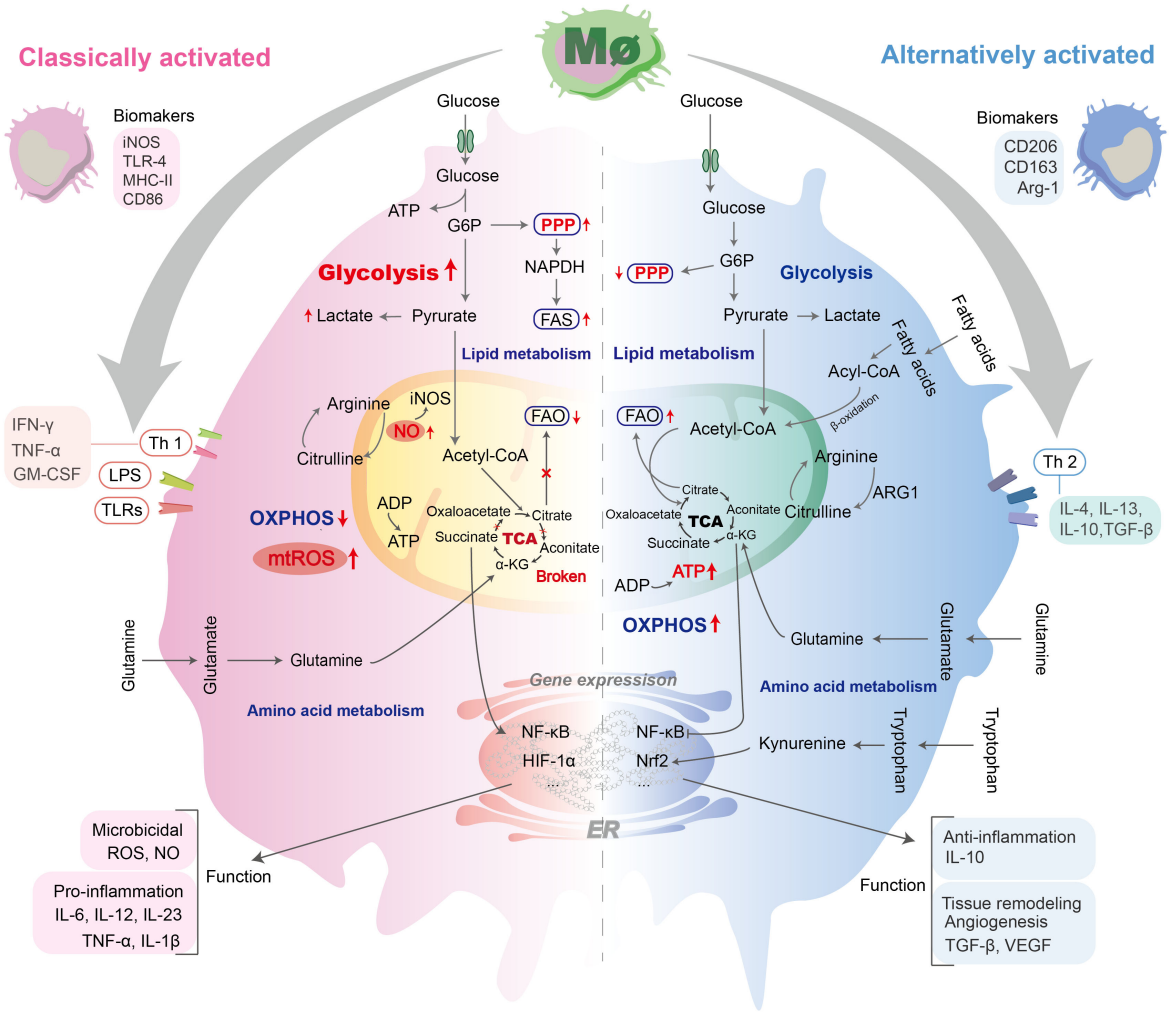
Polarization and metabolic alterations of M1 and M2 macrophages. M1 and M2 macrophages exhibit distinct functional phenotypes shaped by specific stimuli. M1 polarization is driven by LPS, IFN-γ, and TLRs, leading to production of pro-inflammatory mediators (e.g., IL-1β, IL-12, IL-23, ROS, TNF-α). The metabolic changes of M1 macrophages were characterized by enhanced glycolysis, disruption of the TCA cycle, and damage to OXPHOS, accompanied by downregulation of FAO. M2 polarization mainly depended on the complete TCA cycle, OXPHOS, and FAO. M2 polarization is primarily induced by IL-4, IL-13, etc., and characterized by anti-inflammatory/pro-fibrotic responses, exemplified by IL-10 and TGF-β1 release. IL, lnterleukin; MHC-II, major histocompatibility complex II; GM-CSF, granulocyte-macrophage colony-stimulating factor; Acetyl-CoA, acetyl coenzyme A; Acyl-CoA, acyl-coenzyme A; FAO, fatty acid oxidation; Glu, glucose; G6P, Glucose 6-phosphate; OXPHOS, oxidative phosphorylation; ADP, adenosine-diphosphate; ATP, adenosine-triphosphate;α-KG, α-Ketoglutaric acid; ARG1, arginase 1; IFN-γ, interferon γ; TNF-α, tumor necrosis factor-alpha; TGF-β, transforming growth factor-beta; VEGF, vascular endothelial growth factor; LPS, lipopolysaccharide; NO, nitric acid; iNOS, inducible nitric oxide synthase; PPP, pentose phosphate pathway; ROS, reactive oxygen species; mtROS, mitochondrial ROS; TCA, tricarboxylic acid; TLRs, Toll-like receptors.

The concept of immunometabolism was thus introduced to describe how metabolic processes within immune cells influence their functions. Specifically, these processes may not only supply energy for immune responses but also directly shape cellular activity by regulating transcriptional and post-transcriptional events. **Figure 2** outlines the distinct functional states of M1 and M2 macrophages, highlighting their characteristic metabolic reprogramming.

#### 2.2.1 M1 macrophages

M1 macrophage polarization may be induced by metabolic reprogramming toward aerobic glycolysis, a process with potential implications for COPD pathogenesis. This shift is characterized by several interconnected metabolic adaptations that collectively sustain pro-inflammatory responses. 6-phosphofructose-2-kinase/fructose-2, 6-bisphosphatase (PFK2) isomer (L-PFK2) was induced by M1 activation, converted into a more active form (u-PFK2), and led to the accumulation of fructose-2, 6-diphosphate, which promoted intracellular aerobic glycolysis (57). Conversely, the expression of carbohydrate kinase-like protein (CARKL) was inhibited upon the activation of the M1 phenotype, which under basal conditions supports the PPP. The over-expression of CARKL led to the decreased secretion of pro-inflammatory factors, conforming with the M2 phenotype (58). Meanwhile, the increase in the consumption of glutamine and arginine was conducive to exerting the pro-inflammatory function of M1 macrophages (59, 60). Furthermore, LPS-stimulated M1 phenotype metabolism also depends on the interrupted TCA cycle, resulting in the accumulation of several intermediate products, such as citrate, succinate, fumarate, and α-ketoglutarate, with signal transduction functions (53, 61). Among them, succinate was produced by IL-1β, regulated by hypoxia-inducible factor 1-alpha (HIF1α), and the process can be blocked by 2-deoxyglucose via inhibiting glycolysis (62). HIF1α was found by metabolomics to intervene in the expression of multiple glycolytic genes (63) and thus may become a potential target for M1 metabolism.

In brief, we might consider that metabolic events in classically activated macrophages serve a dual purpose: they rapidly provide energy and reduction equivalents to fuel bactericidal activity, and they directly participate in transcriptional regulation to shape immune responses.

#### 2.2.2 M2 macrophages

The low glycolytic levels in IL-4-activated macrophages were reported to be compensated by elevated OXPHOS, and their metabolic activity was achieved by high OXPHOS rates, an intact TCA cycle, and FAO. Activated M2 macrophages massively induced oxidative metabolic programs, including FAO, OXPHOS, and mitochondrial respiration. In the long term, it leads to substantial ATP production via the electron transport chain (ETC) to support cellular anti-inflammatory and repair functions. Simultaneously, constituents of the ETC supported OXPHOS and introduced pyruvate into the Krebs cycle (13, 15, 53). Cellular FA levels were elevated following phagocytosis of apoptotic cells by macrophages, amplifying mitochondrial respiration and inducing NAD+-dependent signaling pathways that triggered anti-inflammatory responses for tissue healing and repair (64). This may imply that FAO is a key metabolic process underpinning M2 macrophage function (65). However, this perspective remains controversial (66), reflecting a critical need to evaluate the strength and context of the evidence. The contradiction primarily centers on whether FAO is an indispensable driver of M2 polarization or a correlative consequence of it. Therefore, while the association between FAO and M2 function is well-supported, its essentiality requires more rigorous validation. Under IL-4 stimulation, the activation of its transcription factor STAT6 induced the secretion of PPARγ-coactivator protein 1β (PGC-1β), which triggered the production of key components of mitochondrial respiration (67, 68), considered critical for the metabolic switch of M2 macrophages. The knockdown of PGC-1β impaired the metabolic profile and functions of the M2 phenotype (69). In addition, PPARs, especially PPARγ and PPARδ, are essential for phenotype maintenance as well, including the coordination of the functions of the alternative activation effect and the transcription of fatty acid β oxidation-related factors (70, 71). Moreover, TNF-α-induced protein 8-like 2 (TIPE2) was also found to induce arginine metabolism (72). Increased Arg1 catalyzed the conversion of arginine to ornithine, which is associated with the M2 polarization phenotype and repair function (13, 61).

The maintenance of M1/M2 polarization balance involves the action of multiple pathways. Inhibiting oxidative metabolism impeded M2 polarization and promoted a shift toward the M1 phenotype. Conversely, forcing oxidative metabolism in M1 macrophages reinforced the manifestation of M2 phenotype (69, 73). Thus, we consider that the state of oxidative metabolism may bidirectionally regulate macrophage polarization. On the other hand, PPP restriction in M2 macrophages led to decreased ROS and NO production, while inhibition of OXPHOS by NO suppressed the polarization from M1 to M2 phenotype under the specific microenvironment (74).

In conclusion, key metabolic diversity in macrophages under different activation states has been widely accepted. Nevertheless, the intricate molecular mechanisms that coordinate these metabolic pathways remain indistinct, and how metabolic patterns specifically regulate the polarization bias has not been fully elucidated.

## 3 Metabolic drivers of macrophage dysfunction in COPD

The increase in both total and polarized AMs may drive COPD pathogenesis through multiple mechanisms, including the secretion of proinflammatory cytokines, chemokines, and MMPs, along with impaired phagocytic and bactericidal functions (75). As mentioned above, the differing polarization trends and cell functions resulting from macrophage metabolic reprogramming may be closely related to the pathogenesis of COPD. In COPD, key immunometabolic features include excessive ROS/NO production, oxidative stress, and iron accumulation. These features are linked to the disease through mitochondrial phenotypic alterations and dysfunction, which appear to play a central mediating role. This connection further elucidates the critical link between macrophage metabolism and COPD pathogenesis. In addition, macrophage-associated glycolysis, amino acid metabolism, and microbial metabolism may also contribute to the pathological mechanism of COPD. Evidences for metabolic alterations of AMs in COPD are summarized in **Table 2**.

**Table 2:** AM metabolic alterations in COPD^#^.

Object	Metabolic change	Functional change and consequence	Ref.
Lung tissues from COPD patients	↑ CD73, ↑ A_2B_R	↑ Adenosine metabolism	(76)
BALF from COPD patients, CD68.hMcl-1-/+ transgenic mice, BMDM	↑ Mcl-1, ↓ Caspase-dependent mROS after pneumococcal challenge	↓ intracellular bacterial killing	(77)
Lung tissues from COPD patients	↓ Gamma-glutamylcysteine synthetase	↓ GSH synthesis, ↑ Oxidative stress	(78)
NO concentration measurement and sputum of COPD patients	↑ iNOS, ↑ Nitrotyrosine	↑ NO production	(79)
BALF and lung tissues from COPD patients	↑ Early mROS, ↓ ΔΨm	Defective bacterial phagocytosis, dysfunctional mitochondria	(80)
BALF and lung tissues from COPD patients	↑ Transferrin, transferrin receptor, and ferritin	↑ Iron sequestration, which may decrease iron-induced oxidative stress	(81)
BALF and sputum from COPD patients, ozone-exposed mice	↑ MIF, ↑ HIF-1α	May increase glycolysis	(82)
BALF from COPD patients	↓ Mitochondrial respiration, ↓ Compensatory glycolysis	Unmet energetic demand, and lower total mitochondrial numbers and mass	(16)
BALF from COPD patients	↓ Coupling efficiency, ↑ Proton leak	↓ OXPHOS, and dysfunctional metabolism	(16)
BALF from COPD patients	Alterations of genes involved in lipid metabolism	Lipid metabolism changes	(83)

^#^ ↑, increased; ↓, decreased; mROS, mitochondrial ROS; ΔΨm, mitochondrial membrane potential; MIF, macrophage migration inhibitory factor.

### 3.1 Mitochondria-associated metabolic reprogramming of macrophages

Mitochondria serve as the central hub for macrophage metabolic plasticity, which plays an important role in energy production, immune regulation, signal transduction, and cell fate determination, intervening the inflammation regulation and macrophage repair (84). A study indicated that AMs in BALF from COPD patients exhibited lower mitochondrial respiration and compensatory glycolytic defects compared with smokers, associated with a lower predicted FEV_1_% (16), supporting results from several prior studies (77, 80, 85). These findings indicated that COPD patients may display abnormal macrophage responses linked to mitochondrial dysfunction. This abnormality may impair the phagocytosis and bactericidal activity of AMs against respiratory pathogens, consequently driving persistent chronic airway inflammation and lung function decline. Mitochondrial metabolism is central to the cellular metabolic network, encompassing the Krebs cycle, OXPHOS, glycolysis, FAO, amino acid metabolism, etc. (86). The intricate communication, whether direct or indirect, between mitochondrial metabolism and AM polarization may lead to changes in cellular function and help explain the shifts in the immunometabolic behavior of AMs within the context of COPD pathology.

#### 3.1.1 ROS and oxidative stress

Chronic exposure to cigarette smoke extracts (CSE) or particulate matter resulted in elevated oxidative stress, a critical metabolic characteristic in AMs of COPD patients (87). Oxidative stress led to a significant increase in the production of mitochondrial reactive oxygen species (mtROS), superoxide anions, and hydrogen peroxide in AMs (88). Moreover, oversecreting of mtROS and a decreased ratio of mROS/superoxide dismutase 2 were linked to defective bacterial killing of AMs (77).

Subsequent research indicated that AMs from COPD patients exhibited impaired phagocytosis associated with mitochondrial dysfunction (80), possibly due to the decreased mitochondrial membrane potential, which caused cellular energy depletion, proton leakage, and overproduction of mtROS (89). Further studies revealed a strong correlation between ROS level and the polarization and metabolic characteristics of macrophages. Typically, mitochondria generate ATP via electron transport and OXPHOS. However, some electrons may escape from protein complex 1 or 3 in the ETC, resulting in the generation of superoxide anion, which is subsequently converted to ROS (such as H_2_O_2_ and hydroxyl radicals·OH). When mitochondria are metabolically active to meet the high metabolic demand, ETC is overloaded, and the likelihood of electron leakage is increased. In addition, upon activation of M1, mitochondria also markedly enhance ROS production via the reverse electron transport (RET) mechanism, thereby facilitating bactericidal and pro-inflammatory functions in cells. However, a high ROS state induces oxidative stress, leading to mitochondrial DNA (mtDNA) damage and membrane integrity disruption. Therefore, the M1 phenotype mainly relies on compensatory glycolysis for rapid energy supply, and mitochondria still maintain part of their function to support the enhanced RET, resulting in the explosively generated ROS (80). On the other hand, a low ROS state shows an OXPHOS-dominated M2 metabolic mode, featured by efficient ETC and reduced ROS production, which is conducive to the maintenance of M2 polarization and facilitates anti-inflammatory and repair functions (90). Thus, ROS plays an important role in the alteration and maintenance of M1/M2 polarization, as well as macrophages’ corresponding functions.

The key pathways of ROS generation are NADPH oxidase (NOX) and RET, which produce pathogen-killing ROS and mtROS, respectively. NOX isoforms 1, 2, 4, and 5 were all observed to remain activated in patients with end-stage COPD, whereas NOX1 and NOX4 mediated oxidative stress and inflammatory responses in mice following acute CS exposure (91). Recently, NOX2 and NOX4 were found to be involved in macrophage polarization related to ROS production. The quantity of NOX2-positive macrophages was elevated in the lungs of emphysema patients, and the elastase-induced emphysema and the high expression of ROS were prevented in NOX2-deficient mice. These findings collectively suggested a potential role of NOX2 in the pathogenesis of emphysema, probably through the sirtuin 1 (SIRT1)/MMP-9 pathway involved in macrophage-specific NOX2 (92). For macrophage polarization, NOX2-dependent ROS generation may be involved in the polarization pathway of primary macrophages to the M2 phenotype, linked to the high-mobility group box 1 (HMGB1)/Toll-like receptor 2 (TLR2)/NOX2 autophagy axis (93). Moreover, NOX4 was identified as a mediator of M1 polarization in mouse intestinal macrophages via ROS (94). Nonetheless, conclusive evidence regarding the role of NOX in AM polarization in COPD remains lacking, highlighting a key area for future research that bridges mechanistic findings to COPD pathology. The ROS degradation program exhibited a correlation with macrophage polarization as well. Peroxiredoxins (Prxs) serve as crucial antioxidant enzymes that eliminate excessive ROS in cells, thereby sustaining redox homeostasis. Deficiency of Prx5 in macrophages can induce M2 polarization and a reduction in M1-related inflammatory factor expression in lung cancer macrophages, while N-acetylcysteine (NAC), an antioxidant, was observed to suppress this tendency by inhibiting ROS production (95).

Mitophagy in macrophages, as a selective autophagy mechanism, may also influence macrophage differentiation through its impact on ROS levels. Under external environments such as ROS stress, nutrient deficiency, and cellular aging, mtDNA mutations accumulate, along with a decrease in mitochondrial membrane potential and depolarization damage (96). Damaged mitochondria are encapsulated into autophagosomes and fused with lysosomes to complete degradation. This process is called mitophagy, specifically removing dysfunctional mitochondria from the cytoplasm to maintain mitochondrial functional integrity and cellular homeostasis (97). Autophagy inhibition was observed to enhance the production of ROS-associated macrophage migration inhibitory factor (MIF), which induced and continuously promoted M1 macrophage polarization (98). Low levels of MAP kinase kinase 3 (MKK3) can enhance mitophagy and regeneration of macrophages, protecting mice from sepsis-induced lung injury (99). In contrast, another study indicated that NIX-dependent mitophagy contributes to the elimination of mitochondria during macrophage polarization to the proinflammatory and more glycolytic M1 phenotype, promoting a metabolic transition toward glycolysis in macrophage metabolism (100). However, the role of mitophagy in macrophage polarization within the context of COPD still requires further investigation.

The AM-related ROS pathway and iron accumulation in COPD have also received attention in recent years. Iron metabolism was revealed to be integral to the ROS pathway. It influenced mtROS production and lipid peroxidation, thereby activating necroptosis and ferroptosis. These forms of cell death contributed to the pathogenesis of COPD (101). With the increasing severity of COPD and emphysema, the amount of iron deposition and the percentage of iron-positive macrophages (AM as the main type) increased. It supported an iron chelation mechanism activated by AMs in COPD, potentially serving as a protective mechanism against iron-induced oxidative stress (81). Based on this, an *in vitro* study found that treating COPD with sulforaphane can activate the antioxidant and anti-inflammatory NRF2 pathway, restoring bacterial recognition and phagocytosis in AMs (102). Moreover, the use of a mitochondrial iron chelator or a low-iron diet can protect mice from CS-induced COPD, jointly supporting the therapeutic potential of targeting the mitochondria-iron axis in COPD (103).

#### 3.1.2 Glucose metabolism

Macrophages can manifest with bidirectional metabolic changes between aerobic OXPHOS and anaerobic glycolysis in response to specific microenvironmental stimuli (40). Early studies in immune cell metabolism identified aerobic glycolysis in neutrophils. It is characterized by increased glucose uptake and an accelerated glycolytic rate despite sufficient oxygen, leading to excessive lactate production and suppressed OXPHOS. This metabolic shift helped to generate the bio-precursors and energy required for cell proliferation (104, 105).

A study on AM metabolic profiles indicated that AMs in COPD exhibited a diminished capacity to dynamically compensate for mitochondrial dysfunction through increasing glycolysis. This pattern manifested with significantly reduced compensatory glycolysis, non-glycolysis, and non-mitochondrial extracellular acidification rate (ECAR) in COPD smokers. The AMs of COPD patients showed impairments in mitochondrial respiration and compensatory glycolysis, correlating with the decreasing trend of lung function (16). For the direction of AM polarization, glycerol-3-phosphate dehydrogenase 2 (GPD2) was found to shift the metabolic pattern of M1 macrophages during infection and promote the activation of the M2 phenotype during tissue repair by modulating glycolysis (106). Mitochondrial dysfunction in lung epithelial cells can lead to defective ATP production and enhanced glycolysis, thereby promoting arsenic-induced massive lactate production and inducing polarization toward the M2 phenotype (107). The above results indicate that glycolysis is closely related to mitochondrial function in AMs of COPD, affecting the activation state and polarization direction of macrophages. Nonetheless, its impact on macrophage function within the COPD environment and the precise mechanisms involved in the disease process remain to be further explored.

#### 3.1.3 Lipid metabolism

Lipids encompass both fats and lipoids, of which metabolic processes are functionally coupled with mitochondria, playing a crucial role in maintaining lung function (108). In COPD, AMs exhibited GOLD-level-dependent lipid metabolism alterations (83), further indicating the potential association between COPD lipid metabolism and the phenotype and function of AMs.

The fatty acid metabolism process involving mitochondria includes fatty acid synthesis (FAS) and FAO. Mitochondria degrade glucose in the cytoplasm via glycolysis, yielding pyruvate, which is then catalyzed by the pyruvate dehydrogenase complex (PDH) to generate acetyl coenzyme A, serving as the crucial raw material for FAS. Acetyl coenzyme A undergoes transmembrane transport through the citrate-pyruvate cycle. FAO refers to the cellular process of fatty acid degradation for energy production. At this time, acyl-coenzyme A enters the mitochondrial matrix and engages in the β-oxidation of long-chain fatty acids within the mitochondria, generating acetyl coenzyme A and reducing equivalents (NADH/FADH_2_), providing energy for the cell and participating in immune regulation (109). The direct effects of the FAS and FAO pathways on macrophage polarization have not been fully elucidated. Nevertheless, studies have indicated that the regulation of their states may coincide with alterations in macrophage phenotype, suggesting possible indirect or potential connections between them. For example, a study on macrophage FAS found that IL-4 can activate sterol regulatory element-binding protein 1 (SREBP1), triggering the *de novo* lipogenesis (DNL) program, separating it from the cell’s antioxidant defense by depleting NADPH, thus increasing ROS levels. In this process, ROS acts as a second messenger, transmitting adequate DNL signals to promote the polarization of the macrophage M2 phenotype (110). On the other hand, FAO may also be related to macrophage polarization. The Fgr tyrosine kinase may promote the M1 polarization phenotype of macrophages via the phosphorylation complex II. Additionally, the clearance of mitochondrial peroxides can inhibit Fgr activation and lead to increased FAO levels (111), indicating that mitochondrial ROS-Fgr kinase may be a key regulatory axis for pro-inflammatory macrophage activation.

The above results indicate that mitochondrial FA metabolism may play a significant role in the polarization of macrophages in COPD. Additionally, sphingolipid metabolism also plays an important role in the pathogenesis of COPD by regulating the function of AMs. Ceramide, a core product of sphingolipid metabolism, can inhibit the endocytosis of AMs by down-regulating Rac1. The excessive accumulation of ceramides may impair the cell skeleton function and consequently diminish the ability of AMs to engulf apoptotic cells, thereby amplifying the damage of emphysema (112). This process may involve its metabolite sphingosine-1-phosphate (S1P), indicating that modulating the production of sphingolipid metabolites could be a viable approach for restoring the phagocytic function of AMs to treat COPD (113). Currently, there is still inadequate evidence to illustrate its impact on the polarization phenotype of COPD macrophages.

#### 3.1.4 Amino acid metabolism

The important role of amino acid metabolism in COPD has been confirmed (114). Studies revealed that the expression of genes related to glutathione metabolism, mitochondrial transport, pyruvate metabolism, the TCA cycle, ETC were altered in smokers and COPD patients (16). Glutathione (GSH) is a tripeptide consisting of three amino acids: glutamic acid, cysteine, and glycine. This important antioxidant can safeguard cells from oxidative damage by neutralizing free radicals. Procysteine, its precursor, has been shown to enhance the phagocytic function of AMs in COPD mice (37). In addition, the expression of iNOS in COPD AMs increased, leading to elevated levels of NO and adenosine receptor A2BR (76, 79). The rise in adenosine metabolism in COPD may be related to the increased level of HIF1α in AMs (82). Moreover, it has been observed that CS can promote the glutamine metabolism of raw cells and induce their M2 polarization phenotype (115). However, the evidence regarding amino acid metabolism’s impact on macrophage polarization and function in COPD is still insufficient and requires further research.

### 3.2 Microbial metabolism

The interaction between the gut microbiome, its metabolites, and the pathophysiology of pulmonary diseases is referred to as the “gut-lung axis”, which has been widely discussed in respiratory diseases (116, 117). The microbial communities colonizing the airways and alveoli, such as proteobacteria, bacteroides, firmicutes, and actinobacteria, can generate numerous metabolites, notably short-chain fatty acids (SCFAs) such as acetic acid, propionic acid, and butyric acid. These metabolites can modulate pulmonary immune-inflammatory responses, affect pulmonary barrier function, and thereby intervene in the disease progression (118). Currently, macrophages are considered the main target of SCFA produced by the microbial community (119).

According to a study by Ji et al., a probiotic mixture enhanced antiviral defense by modulating the gut-lung axis: it increased gut-derived acetate and beneficial lung bacteria (including corynebacterium and lactobacillus), which improve pulmonary microbiome dysbiosis induced by respiratory syncytial virus (RSV), and boost AM phagocytosis and IFN-β secretion. These results highlighted that the microbiome-AM axis may serve as a potential pathway for regulating AM function and its downstream factors (120). Direct evidence from multi-omics analyses of airway host-microbe interactions in COPD patients indicated that indole-3-acetic acid (IAA) derived from airway microbiota alleviated neutrophil inflammation, cell apoptosis, emphysema, and lung function decline through IL-22-mediated macrophage-epithelial cell interactions. And intranasal inoculation of two airway lactobacillus (potential producers of airway IAA) for 6 weeks can alter the lung microbiome in mice, restore IAA levels, and exert a protective effect in COPD mice (121). These studies proposed a potential link between the metabolic activities of the pulmonary microbiome and the functionality of AMs in COPD. However, the effects of SCFA on the metabolism and polarization phenotype of AMs are still in the exploration stage. Studies in experimental models further clarify the roles of specific SCFAs. Butyrate has been found to alter metabolic pathways in macrophages, leading to an increase in OXPHOS and fatty acid metabolism, and inducing polarization towards the M2 phenotype. Although the specific mechanism is still unclear, SCFAs have been reported to induce the activation of genes related to the aforementioned metabolic pathways (122, 123). Furthermore, butyrate and propionate have been reported to inhibit M2 polarization and mitigate inflammatory responses caused by allergic airway reactions in animal models. And the effects of butyrate, butyric acid, and propionate on macrophage lines may be mediated by the activation of GPR43 receptors and/or inhibition of HDAC enzymes (124). These apparent contradictory evidences regarding the pro- versus anti-M2 effects of SCFAs may be due to methodological differences. Key variables such as the specific disease context, the timing and concentration of SCFA exposure, and the source of macrophages may profoundly influence the outcome. The observed dual effects could suggest a context-dependent, homeostatic role for SCFAs in immune responses. Therefore, future research might systematically control these variables to clarify the precise conditions under which SCFAs promote or suppress M2 polarization, which is essential for evaluating their therapeutic potential.

In summary, while the precise mechanisms require further clarification, microbiome-derived metabolites, including IAA, propionate, and butyrate, may intervene in AM function and polarization in COPD. This regulation occurs via the gut-lung axis and within the local pulmonary microenvironment, representing a promising novel therapeutic target.

These evidences in Chapter 3 suggest the potential mechanisms underlying the immune-related metabolic changes in AMs. While direct longitudinal studies tracing the temporal dynamics of AM immunometabolism in human COPD are still emerging, the existing evidences allows for an assumption of this process. In the acute exacerbations or early phases driven by CS, AMs may undergo a metabolic shift towards glycolysis to meet the urgent energy demands for pro-inflammatory responses. This state is characterized by the upregulation of M1-like markers and the release of ROS and proteases, contributing to tissue damage. As COPD transitions to a more chronic phase, the sustained metabolic stress and altered microenvironment may lead to mitochondrial dysfunction and the persistence of inflammation. Notably, some populations of AMs might adopt a mixed or alternative (M2-like) activation state, potentially relying on oxidative phosphorylation to varying degrees, which could paradoxically contribute to impaired bacterial clearance and fibrotic repair processes, thus driving disease progression. Spatially, the metabolic phenotype of AMs is likely shaped by local niches. For instance, the alveolar compartment might favor certain metabolic adaptations due to its direct exposure to inhaled stimuli and unique oxygen tension, whereas the bronchial tree could present a different set of metabolic challenges and inflammatory signals. Future studies could employ single-cell technologies on longitudinally collected patient samples are crucial to validate this spatiotemporal model and uncover novel, stage-specific therapeutic targets.

## 4 Prospective therapies targeting macrophage immunometabolism in COPD

Based on the central role of macrophage immunometabolism in COPD, targeting this axis represents a promising therapeutic strategy. Numerous natural compounds derived from medicinal plants, animals, and fungi, such as flavonoids, terpenoids, and phenols, have shown potential in modulating the immune-metabolic state in pre-clinical models of COPD (125–135), offering the advantages of fewer toxic side effects and multi-targeted mechanisms (136). The potential mechanisms of these compounds can be categorized as follows:

First, certain flavonoids can impact macrophage polarization and abundance. Fisetin, rutin, and casticin were reported to effectively reverse the increase in neutrophil and macrophage populations in BALF of rats/mice induced by CS, mitigating oxidative stress and inflammation in lungs (127, 130, 135). Naringenin, as the main flavanone in citrus, was reported to suppress the disorder of extracellular vesicular cargoes derived from BEAS-2B induced by CSE, thereby inhibiting M1 macrophage polarization (133). Second, a key mechanism involves the enhancement of antioxidant defenses. Fisetin and oroxylin A activated the Nrf2 pathway to alleviate oxidative stress (128, 130), which was similar to the efficacy of ginsenosides (126). Third, some compounds demonstrate the ability to ameliorate mitochondrial dysfunction in macrophages. Andrographolide, a diterpene lactone compound, can alleviate mitochondrial dysfunction, inflammation, and oxidative stress in RAW 264.7 macrophages exposed to CSE by inhibiting SIRT1/ERK signaling, thereby preventing tissue damage in COPD (132). Finally, several compounds showed efficacy in specific, COPD-relevant models. Compound K (CK), a secondary ginsenoside, may act as a potential ligand for glucocorticoid receptors and a regulator of macrophage inflammatory responses, exerting potential effects on COPD (134). Triterpene acids from Eriobotrya japonica (Thunb.) Lindl. Leaf significantly inhibited NO and iNOS in AMs of chronic bronchitis rats, associated with the inhibition of p38 MAPK phosphorylation signal transduction (125). Furthermore, astaxanthin and olive kermes can reduce macrophage infiltration and tissue destruction in the BALF of COPD mice induced by CS (129, 131).

While the aforementioned findings are primarily derived from preclinical models, such as CSE-treated animals or cells, they demonstrate the possibility of using natural products to intervene in COPD pathology by regulating macrophage metabolic imbalances. However, we still lack direct evidence for the specific targets towards AMs. Future studies should consider mechanistic exploration in COPD AM models to advance clinical translation.

Micro-RNAs (miRNAs), as small, endogenous non-coding RNA molecules, are considered key regulators of metabolic homeostasis that influence macrophage function and inflammatory response in chronic lung diseases associated with mitochondrial dysfunction (137, 138). In COPD, the M1 and M2 phenotypes of AMs exhibited distinct miRNA expression profiles, implying their regulatory role in macrophage polarization (139, 140). Moreover, in Mycobacterium tuberculosis (Mtb) infection, Let-7f miRNAs can inhibit NF-κB inhibitor A20 by targeting NF-κB, promoting the polarization of M1 macrophages and NF-κB activity (141). At the same time, let-7adf can promote the expression of pro-inflammatory cytokine IL-6 and the accumulation of glycolysis and succinate through targeting the succinate metabolic pathway in LPS-activated macrophages (142). Let-7a has also been reported to target SNAP23 in colorectal cancer cells, thereby inhibiting OXPHOS (143). These results emphasize the significance of miRNAs in the metabolic reprogramming of macrophages; however, their clinical application in COPD requires a more comprehensive understanding of their mechanisms and targets, which still faces challenges.

On the other hand, targeting specific metabolic pathways may reshape the metabolism and phenotypic changes of COPD macrophages, potentially serving as a strategy to inhibit inflammation. Dimethyl fumaric acid (DMF) functions as a chemical regulator of macrophage phenotype (144). It has been found to inhibit the catalytic cysteine of glycolytic enzyme glyceraldehyde-3-phosphate dehydrogenase (GAPDH), thereby down-regulating aerobic glycolysis in activated myeloid and lymphoid cells, altering the phenotype of macrophages (145). Given that M1 macrophages are characterized by an increased aerobic glycolysis rate and glucose demand, the inhibition of glycolysis may serve as an effective way to manipulate macrophage metabolism and suppress inflammation. 2-Deoxy-D-glucose (2DG), a glycolysis inhibitor, can competitively bind to hexokinase to inhibit glycolysis, subsequently reducing the inflammatory response triggered by M1 macrophages (61). The glycolysis regulator TEPP-46 can inhibit the pro-inflammatory effect of macrophages by obstructing the tetramerization of pyruvate kinase M2 (146) and simultaneously enhance the tolerance of macrophages to endotoxin (147). Moreover, the small molecule CPUY192018 can down-regulate glycolysis in AMs of COPD patients, thereby enhancing their phagocytic function (148).

Based on this, targeted delivery of drugs to AMs may enable the precise regulation of AM immunometabolism, allowing for the optimization of therapeutic effects and avoiding capture by mucus (149). An inhalation-based drug delivery system targeting AMs employs micro- and nanocarriers for implementation. Currently, micro- and nanospheres coated with mannose, which is based on the phagocytic activity of AMs, have been developed for the targeted delivery of antibiotics in the treatment of AM bacterial infections (150). It also provides ideas for drug delivery to AMs in other diseases, such as COPD. As mentioned in this article, chelating agents, antioxidants, miRNAs, chemical regulators, etc., may be able to regulate AM immunometabolism to treat COPD. Incorporating them into aerosolized micro- or nano-delivery systems to assist in drug administration may represent a potential idea for the development of new drugs for COPD.

## 5 Conclusion

In the lungs, macrophages polarize towards the M1/M2 direction under the activation of inflammatory factors, exerting pro-inflammatory/anti-inflammatory effects and participating in the COPD process of airway inflammation, lung parenchymal damage, and repair. They engulf and eliminate apoptotic and necrotic tissue cells, collaboratively shaping the microenvironment of the lungs in COPD.

However, research on the staged induction of macrophage polarization by CS remains insufficient. Further studies are required to clarify the role of AMs in the microenvironment of COPD lungs based on the polarization tendencies and functional states at different disease stages, as well as the specific molecular mechanisms involved in these mechanisms to participate in the disease, so as to provide clear directions for macrophage-targeted COPD treatment. In summary, despite its utility as a foundational framework, the M1/M2 model has limitations in COPD. Future studies may therefore apply techniques such as single-cell RNA sequencing to elucidate the full spectrum of macrophage heterogeneity across COPD stages and subtypes, to deepen the understanding of macrophage phenotypes.

On the other hand, the metabolic reprogramming of macrophages under microenvironment stimuli, involving oxidative stress, mitochondrial dysfunction, and metabolic pathways such as glycolysis, lipogenesis, amino acid metabolism, and microbial metabolism, is closely related to their polarization direction and functional expression, contributing to the pathogenesis of COPD. Interfering with metabolic patterns to reshape the phenotype of macrophages is considered a promising therapeutic strategy, but before this can be achieved, a more accurate understanding of the relationship between metabolic pathways and phenotypes is required. This review explored the mechanisms of metabolic adaptation on AM phenotypes and functions in COPD, highlighting the potential of AM metabolic plasticity for drug development and offering novel concepts for targeted treatment strategies in COPD.

## Glossary

COPD: chronic obstructive pulmonary disease
WHO: World Health Organization
AMs: alveolar macrophages
BALF: bronchoalveolar lavage fluid
M1: classically activated
M2: alternatively activated
OXPHOS: oxidative phosphorylation
ROS: reactive oxygen species
TCA: tricarboxylic acid
TR-: tissue-resident
MO-: monocyte-derived
MDMs: monocyte-derived macrophages
BMDMs: bone marrow-derived macrophages
LPS: lipopolysaccharide
IFN-γ: interferon-γ
iNOS: inducible nitric oxide synthase
IL: interleukin
TNF-α: tumor necrosis factor α
GM-CSF: granulocyte-macrophage colony-stimulating factor
CXCL9/MIG: chemokine (C-X-C motif) ligand 9
CCL2/MCP-1: chemokine (C-C motif) ligand 2
NF-κB: nuclear factor kappa-B
PPARγ: peroxisome proliferator-activated receptor γ
Wnt5a: Wnt family member 5a
TGF-β: transforming growth factor-β
Arg1: arginase 1
MMP: matrix metalloproteinase
PPP: pentose phosphate pathway
FA: fatty acid
FAO: fatty acid oxidation
CARKL: carbohydrate kinase-like protein
HIF1α: hypoxia-inducible factor 1-alpha
PGC-1β: PPARγ-coactivator protein 1β
TIPE2: TNF-α-induced protein 8-like 2
CSE: cigarette smoke extracts
mtROS: mitochondrial reactive oxygen species
ETC: electron transfer chain
RET: reverse electron transport
mtDNA: mitochondrial DNA
NOX: NADPH oxidase
SIRT1: sirtuin 1
HMGB1: high-mobility group box 1
TLR2: Toll-like receptor 2
Prxs: Peroxiredoxins
NAC: N-acetylcysteine
MIF: migration inhibitory factor
MKK3: MAP kinase kinase 3
ECAR: extracellular acidification rate
GPD2: glycerol-3-phosphate dehydrogenase 2
FAS: fatty acid synthesis
PDH: pyruvate dehydrogenase complex
SREBP1: sterol regulatory element-binding protein 1
DNL: *de novo* lipogenesis
S1P: sphingosine-1-phosphate
GSH: Glutathione
SCFA: short-chain fatty acids
RSV: respiratory syncytial virus
IAA: indole-3-acetic acid
miRNAs: Micro-RNAs
Mtb: Mycobacterium tuberculosis
DMF: Dimethyl fumaric acid
GAPDH: glyceraldehyde-3-phosphate dehydrogenase
2DG: 2-Deoxy-D-glucose
